# Clinical and genetic spectrum of factor XII deficiency in the Han population of East China

**DOI:** 10.1186/s13023-024-03404-6

**Published:** 2024-10-09

**Authors:** Fei Xu, Langyi Qin, Anqing Zou, Lingling Hou, Mingshan Wang, Bile Chen

**Affiliations:** 1 Department of Laboratory Medicine, The First Affiliated Hospital of Wenzhou Medical University, Key Laboratory of Clinical Laboratory Diagnosis and Translational Research of Zhejiang Province, Wenzhou, 325015 China; 2 Department of Blood Transfusion, The First Affiliated Hospital of Wenzhou Medical University, Wenzhou, 325015 China

**Keywords:** Hemorrhagic, Factor XII deficiency, F12 gene, Mutation, Thrombophilias

## Abstract

**Background:**

Factor XII (FXII or F12) deficiency is a rare inherited disorder, typically lacking haemorrhagic symptoms. There is limited literature exists on FXII deficiency and mutations within the Chinese population. This study aimed to characterize the spectrum of F12 gene mutations in a Chinese cohort and to investigate the relationship between FXII mutations and clinical phenotypes.

**Methods:**

Genetic and clinical data from 51 unrelated probands with FXII deficiency, along with their families, were meticulously collected and analysed.

**Results:**

Genetic analysis revealed that 94.1% of probands carried genetic defects, with 29 mutations pinpointed in the F12 gene. Of these, 18 mutations were previously reported for the first time by our research group, including c.303_304delCA, c.1078G > A, c.1285 C > T, among others. Of the mutations, 17 are missense, constituting 58.6% of the total. Additionally, 11 are deletions or insertions, of which 8 result in frameshifts, while the remaining one is a nonsense mutation. These mutations were predominantly concentrated in two crucial regions: the catalytic domain and the kringle domain. The most frequently observed mutations were c.1681G > A, closely followed by c.1561G > A and c.1078G > A, indicating a dominance among these mutations. Additionally, a prevalent polymorphism at position 46 was observed in the majority of probands, with 47.1% having the 46T/T genotype and 13.7% having the 46 C/T genotype, which may potentially impact FXII activity. The broad spectrum of asymptomatic FXII deficiency observed within the Han population of East China.

**Conclusions:**

We speculate on the potential impact of recurrent mutations on the efficacy of new drugs being developed to target FXII for thrombosis prevention and treatment. Furthermore, it is important to explore their influence on FXII-related pathways beyond the activation of the contact pathway in the coagulation cascade.

**Supplementary Information:**

The online version contains supplementary material available at 10.1186/s13023-024-03404-6.

## Introduction

Factor XII (FXII or F12), also known as Hageman factor, was first identified by Ratnoff in 1955 in a patient named John Hageman. It is an 80 kDa glycoprotein composed of 596 amino acid residues. The basic structure of FXII protein includes an N-terminal heavy chain featuring fibronectin (FT)-I and FT-II domains, epidermal growth factor (EGF)-I and EGF-II domains, a kringle domain, a proline-rich domain, and a C-terminal light chain with a catalytic domain. The F12 gene is situated on chromosome 5q33-qter and is comprised of 14 exons and 13 introns. Exon 1 encodes a signal peptide, exon 2 encodes a region lacking an identified homologous structure, exons 3 and 4 encode regions akin to the type II fibronectin structure, exons 5 and 7 each encode an EGF domain, exon 6 encodes the fibronectin finger-like structure, and exons 11–14 encode the catalytic domain.

Hereditary FXII deficiency is a rare genetic disorder characterised by abnormal levels of FXII, primarily due to mutations in the F12 gene (GenBank: AF538691.1). This disorder is primarily inherited in a recessive pattern, although dominant inheritance is also possible. It is noteworthy that FXII deficiency typically presents with no symptoms, despite its crucial role in physiological pathways such as the intrinsic coagulation pathway, the kallikrein-kinin system, and the immune response [1–3]. The deficiency is usually detected through routine coagulation testing before surgery or during a health check, as it leads to a notable prolongation of the activated partial thromboplastin time (APTT) [4]. The impact of FXII deficiency on human disease remains somewhat unclear, primarily due to a limited number of patients available for study [5]. Few cases in the literature describe bleeding [6], spontaneous abortion [7, 8], or thrombosis [9], particularly cerebral artery thrombosis [10] and deep venous thromboembolism (DVT) [11]. It may also serve as a potential risk marker for myocardial infarction [12] or hemorrhagic stroke [13]. These findings have rekindled researchers’ interest in FXII due to its role in thrombosis without affecting hemostasis, suggesting FXII as a potential target for the development of safer anticoagulants [14, 15]. Based on the reduction in Factor XII antigen (FXII: Ag) levels, the categorisation is as follows: CRM-, which is cross-reactive material negative (undetectable FXII: Ag); CRM+, which is cross-reactive material positive (normal FXII: Ag); and CRMRed, which is cross-reactive material reduced (reduced FXII: Ag) [16].

To enhance the understanding of FXII deficiency’s impact on human disease, clinical data were collected from 51 unrelated FXII-deficient probands and their relatives. The data were collected over a fifteen-year period from 2009 to the present at the First Affiliated Hospital of Wenzhou Medical University. It includes information on the severity, FXII activity, identified mutations, clinical manifestations, and other relevant details.

## Materials and methods

### Participants

We conducted a retrospective analysis on 51 unrelated probands with FXII deficiency who were included in our study from 2009 to 2024, comprising 25 males and 26 females. The ages of the patients ranged from 0.5 to 82 years. Pedigree investigations were completed for 150 relatives of just 30 unrelated probands. The study involved documenting various details about the subjects, including their age, sex, family history, bleeding history, thromboembolic events and consanguinity. Cases of acquired FXII deficiency and individuals with a history of liver and kidney disease were excluded from the study. The phenotype and genotype data of the probands and their relatives are summarised in supplementary Table 1.

### Blood samples

From 2009 to 2024, we collected peripheral blood samples from 201 individuals suspected of having inherited FXII deficiency. After centrifuging at 3000 rpm for 15 min at 8℃, we collected both Platelet-Poor Plasma(PPP) and blood cells. PPP was used for phenotypic examination, while genomic DNA was extracted from leukocytes using the TIANamp Genomic DNA Kit (TIANGEN, Beijing, China), following the manufacturer’s instructions.

### Coagulation assays

A range of coagulation parameters was measured on a STA-R analyzer (Diagnostica Stago, Asnieres sur Seine, France) using matched commercially available kits. Prothrombin time(PT), APTT and factors VIII, IX, XI and XII were determined using one-stage clotting method. While FXII: Ag levels were measured using an enzyme-linked immunosorbent assay (ELISA) kit (Changfeng, Wenzhou, China). All procedures were carried out in accordance with the manufacturers’ instructions. A total of 150 healthy subjects were recruited for this study as controls, comprising 78 males and 72 females, with an average age of 34 years (range: 19–62 years). None had a history of abnormal bleeding or thrombotic tendencies, nor did they have liver or kidney disease.

### Genetic analysis

All coding exons and adjacent intronic regions of the F12 gene were amplified using polymerase chain reaction (PCR). Primers used for amplification were derived from the published sequence (GenBank accession number: AF538691.1). PCR was performed in a 50-µl reaction volume using an Applied Biosystem 2720 thermal cycler (Applied Biosystems, Foster City, CA, USA). Amplified regions were sequenced at Sunsoon BIO-Technology Corp (Shanghai, China). Mutational sites were confirmed by reverse sequencing after their initial detection. Additionally, the 46 C/T polymorphic site was sequenced in all subjects.

### In silico analysis

The conservation level among Homo sapiens and seven other homologous species: Mus musculus, Rattus norvegicus, Pan troglodytes, Macaca mulatta, Canis lupus familiaris, Bos taurus, and Xenopus tropicalis, was assessed using the ClustalX-2.1-win software(HomoloGene, http://www.ncbi.nlm.nih.gov/homologene). Prediction of mutation pathogenicity and impact was performed using four online bioinformatics tools: PolyPhen-2 (http://genetics.bwh.harvard.edu/pph2/), PROVEAN (http://provean.jcvi.org/seq_submit.php), MutationTaster (http://www.mutationtaster.org) and SIFT (http://sift.jcvi.org/). In addition, PyMOL (http://www.pymol.org), a molecular visualisation software, was used to analyse the spatial structure of FXII before and after the introduction of these mutations.

### In vitro expression

Mutant variants were constructed using the QuikChange kit from Stratagene, with the pIRES2-EGFP/FXII cDNA expression plasmid as a template. Subsequently, COS7 cells were transfected using Polyfect reagent to assess FXII activity and antigen levels. FXII activity in the cell culture supernatant was measured using a coagulation assay, while FXII: Ag in both supernatant and lysate were quantified using ELISA. The expression level of wild-type FXII was set at 100%.

## Results

### Coagulation assays

Laboratory tests for all probands revealed significantly prolonged APTT ratios and markedly decreased levels of Factor XII Coagulant Activity (FXII: C) and FXII: Ag. Other endogenous coagulation factors, such as VIII, IX, and XI, were found to be normal. Some relatives with the same homozygous or compound heterozygous mutations as the probands exhibited nearly identical laboratory test results. Relatives with a heterozygous mutation exhibited normal or only slight prolongation of the APTT ratio, along with decreased levels of FXII: C and FXII: Ag. In contrast, relatives without mutations had normal laboratory test results, whereas those with 46 C/T or 46T/T polymorphisms showed only slight decreases in FXII: C and FXII: Ag levels. Detailed data for the probands and their relatives are summarised in supplementary Table 1.

### Molecular characterization

**Fig. 1:**
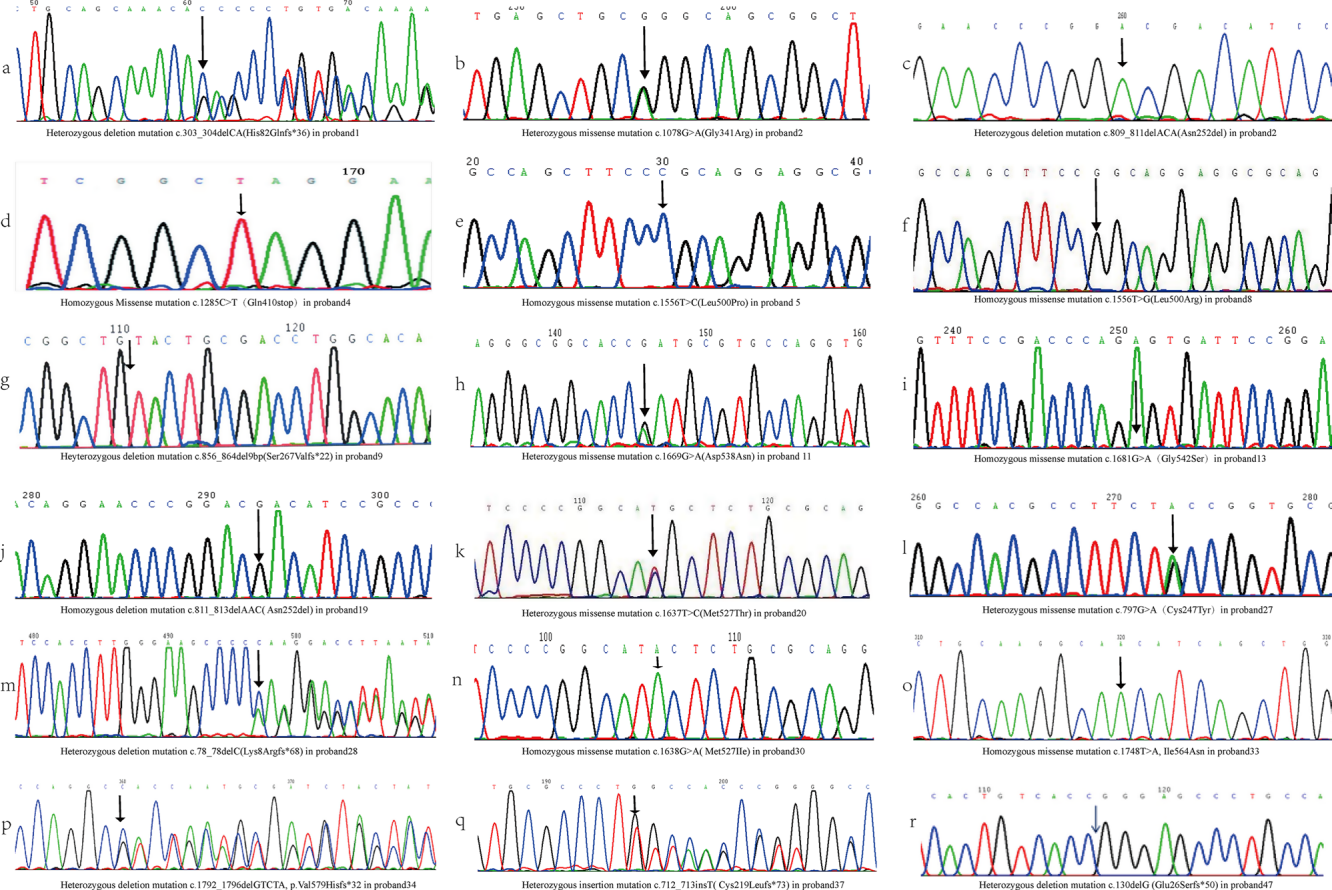
F12 gene sequences of all mutations reported for the first time by our team. The arrow indicates the mutant site

**Fig. 2:**
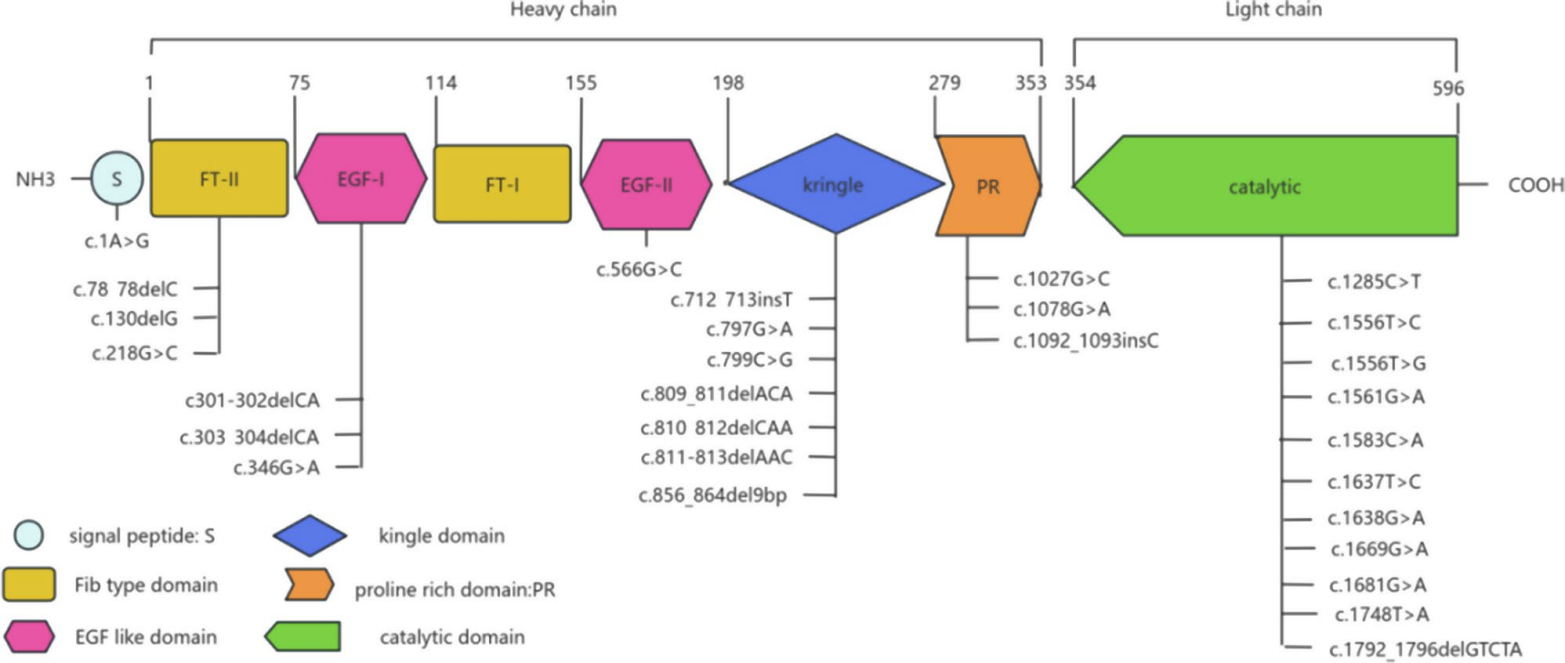
FXII Structural Domain Diagram with all identified mutations

Genetic analysis revealed that 94.1% of 51 probands carried identified mutations. It is noteworthy that no candidate mutations were identified through direct sequencing in three probands with severe FXII deficiency. Of the 51 probands, 16.3% were heterozygous, 44.9% were homozygous, and 30.6% were compound heterozygous for the mutations. Among them, 24(47.1%) had the 46T/T polymorphism, and 7(13.7%) had the 46 C/T polymorphism. Surprisingly, all probands with heterozygous, compound heterozygous, or homozygous mutations exhibited severe FXII deficiencies, leading to significantly reduced FXII: C and FXII: Ag levels. All probands in our study were classified as having CRM- FXII deficiencies. In contrast, CRMRed FXII deficiencies were readily identified in their relatives, with no instances of CRM + FXII deficiencies observed. Genetic testing identified 29 distinct mutations in the F12 gene, including 17 missense mutations, 9 deletion mutations, 2 insertion mutations, and 1 nonsense mutation. The detailed data of the probands and their relatives were summarized in supplementary Table 1. Among these mutations, eighteen (e.g., c.303_304delCA, c.1078G > A, c.1285 C > T, and others) were first reported by our team, with their F12 gene sequences depicted in Fig. 1. Figure 2 demonstrates that the identified mutations were distributed almost throughout the F12 gene, affecting various domains including the signal peptide, FT-II domain, EGF-I and EGF-II domains, kringle domain, proline-rich domain, and catalytic domain. We observed that the majority of mutations were missense mutations, followed by deletions, with frameshifts occurring frequently. Eight of the eleven deletion or insertion mutations resulted in frameshifts. Notably, The frameshift mutation Arg2Tyrfs*19, occurring within the promoter region, is of particular interest. The mutation profiles are summarised in Table 1. Most of the identified potential mutations were situated at conserved positions, consistent with those in the F12 gene of homologous species. The majority of mutations were predicted to be pathogenic and to affect protein function. Analysis of the 3D protein model indicated that mutations can either increase or decrease hydrogen bonds, thereby affecting the stability of the peptide chains. Alternatively, they may cause the loss or insertion of amino acids due to frameshifts, leading to protein truncation. These alterations disrupt the spatial conformation of the proteins, consequently impacting the activity of FXII.

**Table 1:** The 29 identified mutations and in silico analysis

Mutation site	amino acid	Exon	Domain	Type of mutation	Frameshift	conservation analysis	PolyPhen-2 Prediction score	PEOVEAN score	Mutation Taster	SIFT	PyMOL
c.303_304delCA	His82Glnfs*36	E5	EGF-I	Deletion	YES	Highly	/	/	disease causing/1	/	/
c.1078G > A	Gly341Arg	E10	Proline-rich	Missense	NO	Highly	0.934	-6.214	disease causing/0.976	0.01	
c.809_811delACA	Asn252del	E9	Kringle	Deletion	NO	Highly	/	/	disease causing/0.932	/	/
c.1561G > A	Glu502Lys	E13	catalytic	Missense	NO	Completely	0.971	-3.760	disease causing/0.991	0.02	YES
c.1285 C > T	Gln410stop	E11	catalytic	Nonsense	NO	Highly	/	/	disease causing/1	/	/
c.1556T > C	Leu500Pro	E13	catalytic	Missense	NO	Highly	1.0	/	disease causing/0.999	0.00	/
c.1681G > A	Gly542Ser	E14	catalytic	Missense	NO	Highly	1.0	-4.975	disease causing/0.999	0.00	/
c.1556T > G	Leu500Arg	E13	catalytic	Missense	NO	Highly	0.998	-5.448	disease causing/0.999	0.00	/
c.856_864del9bp	Ser267Valfs*22	E9	Kringle	Deletion	NO	Moderately	/	/	Polymorphism/0.969	/	/
c.1669G > A	Asp538Asn	E13	catalytic	Missense	NO	Highly	0.998	/	disease causing/0.997	0.00	/
c.811-813delAAC	Asn252del	E9	Kringle	Deletion	NO	Highly	/	/	disease causing/0.951	/	/
c.1637T > C	Met527Thr	E13	catalytic	Missense	NO	Highly	0.999	-5.635	disease causing/0.999	0.00	YES
c.797G > A	Cys247Tyr	E8	Kringle	Missense	NO	Highly	0.995	-9.35	disease causing/0.999	0.00	/
c.78_78delC	Lys8Argfs*68	E2	FT-II	Deletion	YES	Moderately	/	/	disease causing/1	/	/
c.1583 C > A	Ser509Tyr	E13	catalytic	Missense	NO	Weekly	0.710	-2.994	Polymorphism/0.999	0.00	YES
c.1027G > C	Ala324pro	E10	Proline-rich	Missense	NO	Weekly	0.11	/	Polymorphism/0.999	0.03	/
c.1638G > A	Met527ILe	E13	catalytic	Missense	NO	Highly	0.999	-3.74	disease causing/0.999	0.00	YES
c.1092_1093insC	Lys346Glnfs*69	E10	Proline-rich	Insertion	YES	Completely	/	/	disease causing/1	0.00	YES
c.1748T > A	Ile564Asn	E14	catalytic	Missense	NO	Highly	1	-5.320	Polymorphism/0.999	0.00	YES
c.1792_1796delGTCTA	Val579Hisfs*32	E14	catalytic	Deletion	YES	Highly	/	/	disease causing/0.999	/	YES
c.1 A > G	Arg2Tyrfs*19	E1	Signal peptide	Missense	YES	Moderately	0.680	/	disease causing/1	0.02	YES
c.712_713insT	Cys219Leufs*73	E8	Kringle	Insertion	YES	Highly	/	/	disease causing0.986	0.00	YES
c.218G > C	Cys54Ser	E4	FT-II	Missense	NO	/	1	/	disease causing/1	0.00	/
c301-302delCA	His82Glnfs*36	E5	EGF-I	Deletion	YES	Highly	/	/	disease causing/1	/	/
c.799 C > G	Arg248Gly	E8	Kringle	Missense	NO	Highly	/	/	disease causing/0.695	0.00	/
c.566G > C	Cys170Ser	E7	EGF-II	Missense	NO	Highly	/	-7.38	disease causing/0.99	0.00	YES
c.130delG	Glu26Serfs*50	E3	FT-II	Deletion	YES	Moderately	/	/	disease causing/1	/	/
c.346G > A	Gly97Ser	E5	EGF-I	Missense	NO	Highly	0.55	-0.691	Polymorphism/0.863	0.82	/
c.810_812delCAA	Asn252del	E9	Kringle	Deletion	NO	Highly	/	/	disease causing/0.695	/	/

1.PolyPhen-2: the scores are evaluated as 0.000 (most probably benign) to 1.000 (most probably damaging)

2. PROVEAN: the score ≤ − 2.5 (deleterious), >−2.5 (neutral)

3. Mutation Taster: The closer the value is to 1, the more reliable the prediction is

0.4.SIFT: scores range from 0 to 1, ≤ 0.05 (damaging), > 0.05 (tolerated/acceptable)

### Recurrent mutations

**Fig. 3:**
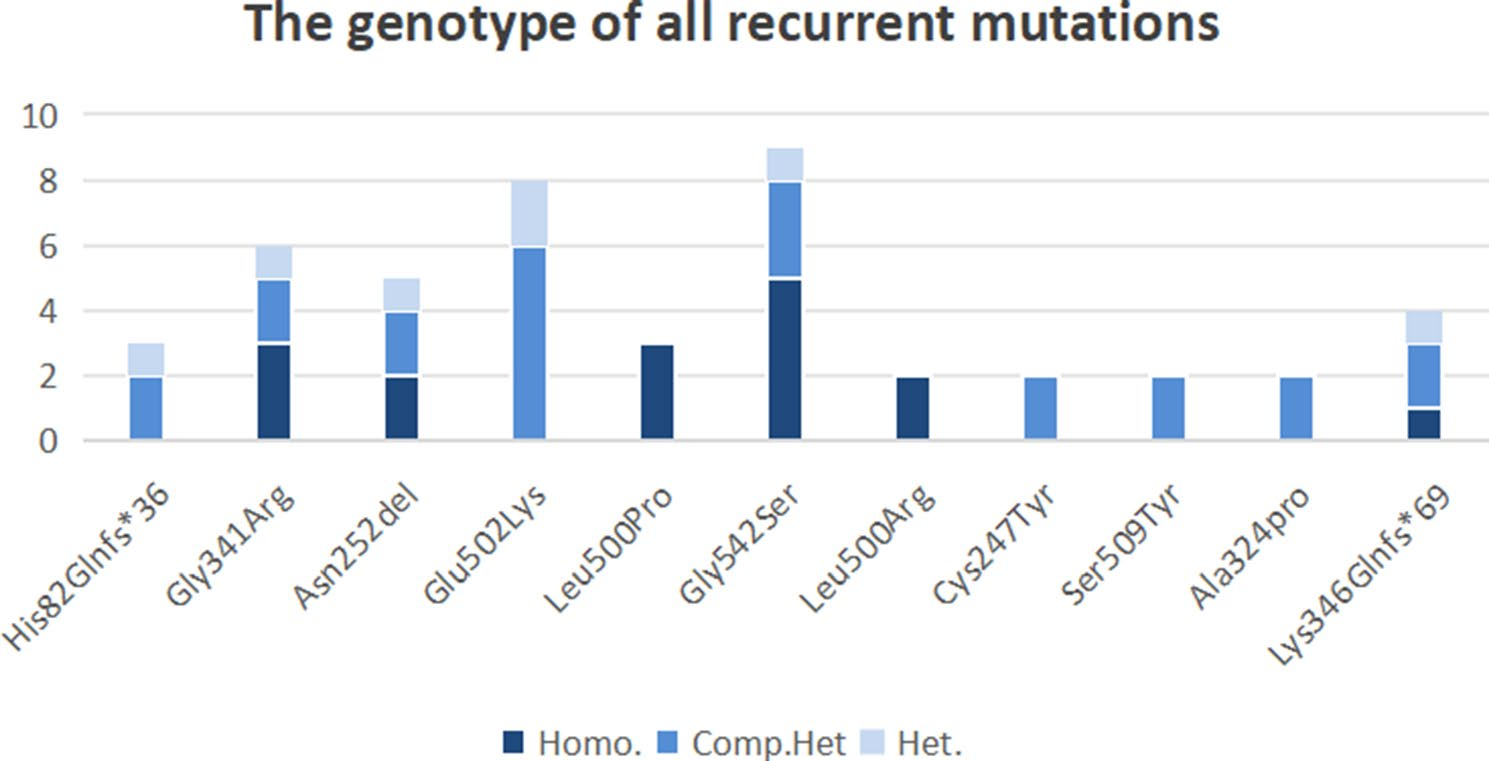
The genotype of all recurrent mutations. The X-axis represents recurrent mutations, while the Y-axis represents the number of probands

Figure 3 presents eleven recurrent mutations, comprising nine missense mutations, two deletions, and one insertion mutation, all observed two or more times during the study. The most prevalent mutation observed was c.1681G > A (Gly542Ser), identified in nine probands, followed by c.1561G > A (Glu502Lys) in eight probands, and c.1078G > A (Gly341Arg) in six probands. Gly542 and Glu502 are situated within the catalytic domain of the light chain, with Gly341 in close proximity. Furthermore, mutations c.1556T > C (Leu519Pro) in three probands and c.1556T > G (Leu519Arg) in two probands were observed. Both mutations involve changes at nucleotide position 1556, where thymine (T) is replaced either by cytosine (C) or guanine (G), resulting in two distinct altered proteins. Similarly, mutations at adjacent sites, such as c.1637T > C and c.1638G > A or c.797G > A and c.799 C > G, may result in the production of distinct proteins. Deletions at adjacent site may result in the production of same proteins, such as c.301-302delCA or c.303_304delCA (His82Glnfs*36) and c.809_811delACA, c.810_812delCAA, or c.811-813delAAC (Asn252del), are also common. Thus, these mutations are recurrent among Chinese individuals with FXII deficiency, potentially due to a founder effect.

### Clinical aspects

The majority of probands were referred for preoperative screening (45.1%) and check-up/outpatient visit (54.9%). At least 11 consanguineous marriages were identified in 30 family studies, and furthermore, 130 out of 150 relatives (86.6%) were confirmed to have the same disease. Detailed data can be found in supplementary Table 1. Proband 31 and 43 do not experience spontaneous bleeding but have difficulty stemming bleeding after trauma. Proband 44 experiences heavy menstrual bleeding, unrelated to gynaecological conditions such as adenomyosis. The bleeding worsened after childbirth and is accompanied by the passing of blood clots. Considering that factor XII deficiency is recognized not to be associated with an increased risk of bleeding, we considered excluding all other potential causes of bleeding, such as other hemostatic defects like von Willebrand factor (VWF) deficiency or platelet function abnormalities. The VWF was assessed on a STA-R analyzer using the immuno-turbidimetric method. The test results indicated that the VWF antigen level of proband 44 was 48% (normal range: 75–125%), while the results for the other two were normal. The routine blood test was conducted using a Mindray BC 6800 PLUS analyser, with results showing that the platelet counts were all within the normal range. The few suspected bleeding manifestations described in this group of FXII deficient individuals (e.g., menorrhagia, hemorrhagic disorder) may be occasional findings unrelated to this coagulation defect. Proband 33, a 27-year-old female patient, presented to our hospital for treatment following shoulder trauma and cervical disc herniation resulting from a car accident. The shoulder injury was treated with five stitches and showed no signs of abnormal bleeding. However, the patient was readmitted 10 days later due to oedema in the lower limbs and secondary varicose veins. Lower limb venography revealed a thrombus in the inferior vena cava and right common iliac vein. Proband 48, a 25-year-old female, has been diagnosed with bilateral tubal obstruction, while Proband 49, a 34-year-old female, is afflicted by severe endometriosis. Both individuals have undergone two unsuccessful in vitro fertilisation and embryo transfer procedures. The other probands were all asymptomatic. Notably, there have been no observations of spontaneous bleeding, even during major surgical procedures.

### In vitro expression

As shown in Table 2, we have successfully completed the in vitro expression of p.Ser267Valfs*22, p.Asp538Asn, p.Glu502Lys, and p.Gly542Ser. In the in vitro expression study, p.Ser267Valfs*22, p.Asp538Asn, and p.Gly542Ser exhibited significantly decreased FXII: C and FXII: Ag levels in the supernatant, confirming secretion defects in the mutant protein. In contrast, p.Glu502Lys exhibited decreased FXII: C and FXII: Ag levels both in the supernatant and cell lysates, indicating defects in both synthesis and secretion of the mutant protein.

**Table 2:** Results of COS7 cells FXII: Ag and FXII: C

Plasmid	Conditioned media FXII:Ag(%)	Conditioned media FXII:C(%)	Cell lysates FXII:Ag(%)
p.Ser267Valfs*22 mutant /Wild	51.9/100	56.4/100	85.6/100
p.Asp538Asn mutant /Wild	35.8/100	30.5/100	90.6/100
p.Glu502Lys mutant /Wild	24/100	28/100	39/100
p.Gly542Ser mutant /Wild	26.5/100	31.3/100	86.8/100

## Discussion

Hereditary FXII deficiency differs from other coagulation factor deficiencies in that it typically does not present with bleeding symptoms. Most individuals may be asymptomatic and are only discovered to have lower levels of FXII: C and FXII: Ag during routine blood tests [17]. Usually, genetic testing and mutation identification can often assist in diagnosis and genetic counselling for factor deficient patients. Although there is no proven benefit of such a strategy across international guidelines for FXII deficiency, since it is not linked to an increased risk of bleeding. Analysing mutations associated with FXII deficiency has demonstrated clear benefits in the development of new drugs for thrombosis prevention and treatment.

As of April 2024, the Human Gene Mutation Database(HGMD, http://www.hgmd.

cf.ac.uk/ac/gene.php? gene = FXII) has recorded approximately 80 mutations of the F12 gene. Most known mutations in F12 gene are missense mutations, while insertional mutations and deletional mutations are less common. Our observation aligns with data in the HGMD, however, it is worth noting that deletions or insertions leading to frameshift mutations occur with notable frequency. The presence of c.1681G > A, c.1561G > A, and c.1078G > A in multiple pedigrees suggests that these mutations may be relatively common in the Chinese population. It is noteworthy that, in addition to the recurrent mutations, the occurrence of mutations at adjacent sites, such as c.1637T > C and c.1638G > A; c.797G > A and c.799 C > G; c.301-302delCA and c.303_304delCA; c.809_811delACA, c.810_812delCAA, and c.811-813delAAC, is quite common, suggesting a potential hotspot for mutations in the F12 gene within the Chinese population. In Chinese pedigrees with FXII deficiency, mutations are more frequently observed in the catalytic domain and kringle region, suggesting these are key regions for FXII mutations within this population. The PyMOL analysis reveals that mutations affect hydrogen bonds, induce amino acid changes, and disrupt protein conformation, thereby impacting FXII activity. In vitro expression experiments confirmed defects in the synthesis and/or secretion of the mutant protein. This suggests that these mutations could significantly alter the biological function of the FXII protein, ultimately affecting its activity. However, three probands with severe deficiency showed no candidate mutations, highlighting the limitations of direct sequencing. This underscores the need for a more comprehensive approach such as next-generation sequencing for genetic testing, which can detect a wider range of mutations, including those that may be more difficult to identify through traditional methods.

Most probands exhibited homozygous or compound heterozygous mutations, which may not accurately reflect the mutation spectrum in the broader population, likely due to the method of case identification. Calculating the true frequency of this defect is challenging because individuals, even those with the most severe forms, are often asymptomatic and are only occasionally discovered during routine laboratory tests due to significant prolongation of the APTT ratio. Further research revealed a significant decrease in FXII: Ag and FXII: C levels, all classified as CRM- FXII deficiency. No instances of CRM + FXII deficiency were identified. In fact, very few reports ([1, 19] relate to type CRM + where gene abnormalities lead to structural abnormalities in FXII. It is worth noting that we also observed ten CRM- FXII deficient probands with a single heterozygous mutation. Among them, seven shared a common 46T/T polymorphism, while the other three had a 46 C/T polymorphism, which may have contributed synergistically to the observed deficiency ([20]. Additionally, CRMRed individuals were found exclusively among family members. They may exhibit only slight or normal prolongation of the APTT ratio, all of whom carried only a single heterozygous mutation. A thorough family study is crucial for identifying individuals who may have inherited CRMRed FXII deficiency, thus preventing the oversight of this condition. These findings suggest that there could be more asymptomatic FXII deficient patients within this population.

Although most of the detected mutations are predicted to be harmful, the majority of carriers remain asymptomatic. The few clinical manifestations observed in individuals with FXII deficiency, such as menorrhagia and post-traumatic lower limb DVT, may be incidental findings unrelated to this coagulation defect. Coincidentally, among these six rare clinically manifested cases, we observed two instances of the c.809_811delACA mutation and two instances of the c.1092_1093insC mutation.

## Conclusions

The mutations, particularly these recurrent ones observed in Chinese individuals with FXII deficiency, prompt us to speculate on their potential impact on the efficacy of novel drugs targeting FXII for the prevention and treatment of thrombosis. Furthermore, it is essential to explore their influence on pathways involving FXII beyond the activation of the contact pathway of the coagulation cascade.

## Electronic supplementary material

Below is the link to the electronic supplementary material.

Supplementary Material 1

Supplementary Material 2

## Data Availability

All data generated or analysed during this study are included in this published article [and its supplementary information files].

## Abbreviations

FXII or F12: Factor XII
FT: Fibronectin
EGF: Epidermal Growth Factor
PT: Prothrombin time
APTT: Activated partial thromboplastin time
DVT: Deep venous thromboembolism
FXII Ag: Factor XII Antigen
CRM-: Cross-reactive material-negative
CRM+: Cross-reactive material-positive
CRM Red: Cross-reactive material-reduced
PPP: Platelet-Poor Plasma
ELISA: Enzyme-linked immunosorbent assay
PCR: Polymerase chain reaction
FXII C: Factor XII Coagulant Activity
