# Supplementary material for: Clinical and genetic spectrum of factor XII deficiency in the Han population of East China
Source: Orphanet J Rare Dis. 2024 Oct 9;19:372. doi: 10.1186/s13023-024-03404-6 (PMC11465813; doi:10.1186/s13023-024-03404-6)
Supplement: Supplementary file 2 — Supplementary Material 2 [file 13023_2024_3404_MOESM2_ESM.docx]

| **P** | **G/A** | **Coagulation test results** | | | | **Mutation site** | **genotype** | **c.-4 genotype** | **Reason for Visit** | **carriers/families** | **consanguinuous marriage** |
| --- | --- | --- | --- | --- | --- | --- | --- | --- | --- | --- | --- |
|  |  | **INR** | **APTTR** | **FXII:C(%)** | **FXII:Ag(%)** |  |  |  |  |  |  |
| 1-Ⅱ2 | M/67 | 0.92 | 2.20 | 2 | <1 | c.303_304delCA▲  c.1092_1093insC | Comp.Het | 46T/T | Preoperative gallstones | 4/9 | NO |
| 1-Ⅱ1 | M/69 | 1.06 | 1.01 | 39 | 32 | wild | / | 46T/T | / |  |  |
| 1-Ⅱ4 | M/65 | 0.96 | 2.35 | 3 | <1 | c.303_304delCA  c.1092_1093insC | Comp.Het | 46T/T | / |  |  |
| 1-Ⅲ2 | M/44 | 0.94 | 1.17 | 29 | 30 | c.1092_1093insC | Het. | 46T/T | / |  |  |
| 1-Ⅲ3 | M/42 | 0.96 | 1.03 | 34 | 37 | c.1092_1093insC | Het. | 46T/T | / |  |  |
| 1-Ⅲ5 | F/43 | 0.92 | 0.9 | 89 | 95 | wild | / | 46C/T | / |  |  |
| 1-Ⅳ1 | F/17 | 0.90 | 1.04 | 94 | 92 | wild | / | 46C/T | / |  |  |
| 1-Ⅳ2 | F/15 | 1.05 | 1.04 | 118 | 115 | wild | / | 46C/T | / |  |  |
| 1-Ⅳ3 | F/11 | 1.00 | 1.03 | 71 | 79 | wild | / | 46T/T | / |  |  |
| 2 | F/47 | 0.91 | 2.68 | 2 | 5.2 | c.1078G>A▲  c.809_811delACA▲ | Comp.Het | 46C/C | Preoperative tooth extraction | / | NO |
| 3 | M/82 | 1.04 | 2.21 | 2 | 1 | c.1078G>A  c.1561G>A | Comp.Het | 46C/C | Preoperative ureteral calculi | / | NO |
| 4-Ⅱ1 | M/34 | 0.83 | 2.81 | 2 | 6 | c.1285C>T▲ | Homo. | 46C/C | Preoperative appendicitis | 4/5 | YES |
| 4-Ⅱ2 | F/33 | 0.77 | 0.89 | 101 | 92 | wild | / | 46C/C | / |  |  |
| 4-Ⅰ1 | F/59 | 0.78 | 1.09 | 52 | 63 | c.1285C>T | Het. | 46C/C | / |  |  |
| 4-Ⅰ2 | M/58 | 0.80 | 1.06 | 62 | 65 | c.1285C>T | Het. | 46C/C | / |  |  |
| 4-Ⅲ1 | M/9 | 0.78 | 1.04 | 59 | 60 | c.1285C>T | Het. | 46C/C | / |  |  |
| 5-Ⅱ1 | F/76 | 0.82 | 3.97 | 0.4 | 4 | c.1556T>C▲ | Homo. | 46C/T | Check up | 3/6 | NO |
| 5-Ⅱ2 | M/73 | 0.78 | 0.98 | 111 | 98 | wild | / | 46C/T | / |  |  |
| 5-Ⅲ1 | M/53 | 0.85 | 1.07 | 56 | 70 | c.1556T>C | Het. | 46T/T | / |  |  |
| 5-Ⅲ2 | M/51 | 0.78 | 1.04 | 61 | 71 | c.1556T>C | Het. | 46C/C | / |  |  |
| 5-Ⅲ3 | F/50 | 0.79 | 0.89 | 101 | 99 | wild | / | 46C/C | / |  |  |
| 5-Ⅳ1 | M/25 | 0.78 | 1.08 | 98 | 95 | wild | / | 46C/C | / |  |  |
| 6-Ⅳ2 | F/48 | 0.86 | 3.23 | 2 | 1 | c.1681G>A | Homo. | 46T/T | Preoperative hemorrhoids | 5/6 | YES |
| 6-Ⅳ3 | M/50 | 0.93 | 0.96 | 108 | 100 | wild | / | 46C/C |  |  |  |
| 6-Ⅲ1 | M/70 | 0.89 | 1.36 | 18 | 13 | c.1681G>A | Het. | 46T/T | / |  |  |
| 6-Ⅲ2 | F/71 | 1.00 | 1.05 | 48 | 40 | c.1681G>A | Het. | 46C/T | / |  |  |
| 6-Ⅳ1 | M/46 | 1.03 | 2.81 | 2 | 1 | c.1681G>A | Homo. | 46T/T | / |  |  |
| 6-V1 | M/23 | 0.99 | 1.06 | 45 | 41 | c.1681G>A | Het. | 46C/T | / |  |  |
| 7-Ⅱ1 | F/0.5 | 0.88 | 2.72 | <1 | <1 | c.1681G>A | Het. | 46T/T | Preoperative lipomyoma | 2/3 | NO |
| 7-Ⅰ1 | M/28 | 0.97 | 1.62 | 21 | 25 | c.1681G>A | Het. | 46T/T | / |  |  |
| 7-Ⅰ2 | F/26 | 0.90 | 1.12 | 51 | 62 | wild | / | 46T/T | / |  |  |
| 8-Ⅰ1 | M/80 | 0.93 | 2.96 | 2 | 1 | c.1556T>G▲ | Homo. | 46T/T | Preoperative hernia | 5/6 | NO |
| 8-Ⅰ2 | F/78 | 0.88 | 0.99 | 128 | 118 | wild | / | 46C/C | / |  |  |
| 8-Ⅱ1 | M/53 | 0.89 | 1.07 | 23 | 21 | c.1556T>G | Het. | 46C/T | / |  |  |
| 8-Ⅱ2 | F/51 | 0.91 | 1.09 | 23 | 23 | c.1556T>G | Het. | 46C/T | / |  |  |
| 8-Ⅱ3 | F/48 | 1.01 | 1.19 | 24 | 23 | c.1556T>G | Het. | 46C/T | / |  |  |
| 8-Ⅲ1 | F/20 | 1.03 | 1.21 | 23 | 23 | c.1556T>G | Het. | 46C/T | / |  |  |
| 9-Ⅱ1 | F/67 | 0.98 | 3.00 | 1 | 1 | c.856_864del9bp▲ | Het. | 46T/T | pesticide poisoning | 4/8 | NO |
| 9-Ⅲ2 | M/42 | 0.87 | 1.17 | 24 | 27 | Wild | / | 46T/T | / |  |  |
| 9-Ⅲ3 | F/40 | 1.01 | 1.34 | 14 | 15 | c.856_864del9bp | Het. | 46T/T | / |  |  |
| 9-Ⅲ4 | F/38 | 0.96 | 1.28 | 15 | 21 | c.856_864del9bp | Het. | 46T/T | / |  |  |
| 9-Ⅳ1 | F/14 | 1.00 | 0.96 | 57 | 62 | wild | / | 46C/T | / |  |  |
| 9-Ⅳ2 | F/10 | 0.98 | 1.06 | 35 | 39 | wild | / | 46T/T | / |  |  |
| 9-Ⅳ3 | F/11 | 1.03 | 1.24 | 16 | 21 | c.856_864del9bp | Het. | 46T/T | / |  |  |
| 9-Ⅳ4 | F/7 | 1.09 | 1.04 | 34 | 32 | wild | / | 46T/T | / |  |  |
| 10-Ⅱ1 | M/35 | 0.94 | 4.38 | <1 | <1 | c.1681G>A | Homo. | 46T/T | Hemoptysis | 4/5 | YES |
| 10-Ⅰ2 | F/59 | 0.90 | 0.93 | 50 | 48 | c.1681G>A | Het. | 46T/T | / |  |  |
| 10-Ⅱ2 | F/33 | 0.90 | 1.12 | 51 | 42 | c.1681G>A | Het. | 46T/T | / |  |  |
| 10-Ⅲ1 | M/10 | 0.97 | 1.34 | 21 | 37 | c.1681G>A | Het. | 46T/T | / |  |  |
| 10-Ⅲ2 | F/6 | 0.88 | 1.06 | 21 | 32 | c.1681G>A | Het. | 46T/T | / |  |  |
| 11-Ⅰ1 | M/60 | 1.01 | 3.38 | 5.3 | 6.8 | c.1669G>A▲ | Het. | 46T/T | Preoperative multiple injuries | 2/6 | NO |
| 11-Ⅰ2 | F/55 | 0.97 | 1.01 | 103 | 101 | wild | / | 46C/T | / |  |  |
| 11-Ⅱ2 | F/34 | 0.95 | 1.06 | 86 | 88.6 | wild | / | 46C/T | / |  |  |
| 11-Ⅱ3 | M/28 | 0.99 | 2.74 | 9 | 12.2 | c.1669G>A | Het. | 46T/T | / |  |  |
| 11-Ⅱ4 | F/26 | 1.00 | 1.16 | 64 | 79.6 | wild | / | 46T/T | / |  |  |
| 11-Ⅲ1 | M/6 | 1.02 | 1.17 | 60 | 70.3 | wild | / | 46C/T | / |  |  |
| 12 | F/27 | 1.07 | 3.16 | 4 | 3 | c.303-304delCA | Het. | 46T/T | Antenatal check-up | / | NO |
| 13-Ⅱ3 | M/38 | 0.96 | 2.83 | 3 | <1 | c.1681G>A▲ | Homo. | 46C/C | Cough | 7/7 | YES |
| 13-Ⅰ1 | M/67 | 0.99 | 1.19 | 29 | 28 | c.1681G>A | Het. | 46C/C | / |  |  |
| 13-Ⅰ2 | F/64 | 1.03 | 1.1 | 35 | 32.4 | c.1681G>A | Het. | 46C/C | / |  |  |
| 13-Ⅱ1 | M/42 | 0.91 | 1.24 | 33 | 30.7 | c.1681G>A | Het. | 46C/C | / |  |  |
| 13-Ⅱ2 | F/40 | 0.98 | 1.12 | 52 | 50.6 | c.1681G>A | Het. | 46C/C | / |  |  |
| 13-Ⅲ1 | M/10 | 1.16 | 1.46 | 25 | 25.2 | c.1681G>A | Het. | 46C/C | / |  |  |
| 13-Ⅲ2 | F/7 | 0.92 | 1.29 | 31 | 30.3 | c.1681G>A | Het. | 46C/C | / |  |  |
| 14-Ⅳ2 | F/26 | 0.92 | 1.71 | 12 | 10 | c.1078G>A | Homo. | 46C/C | Antenatal check-up | 8/10 | YES |
| 14-Ⅱ1 | F/73 | 0.90 | 1.05 | 34 | 32 | c.1078G>A | Het. | 46C/C | / |  |  |
| 14-Ⅱ2 | F/72 | 0.86 | 1.05 | 36 | 35 | c.1078G>A | Het. | 46C/C | / |  |  |
| 14-Ⅲ1 | F/52 | 0.94 | 1.08 | 34 | 37 | c.1078G>A | Het. | 46C/C | / |  |  |
| 14-Ⅲ2 | M/50 | 1.02 | 1.06 | 35 | 30 | c.1078G>A | Het. | 46C/C | / |  |  |
| 14-Ⅲ3 | F/47 | 0.99 | 0.99 | 99 | 97 | wild | / | 46C/C | / |  |  |
| 14-Ⅲ4 | M/47 | 0.90 | 1.02 | 98 | 95 | wild | / | 46C/C | / |  |  |
| 14-Ⅲ5 | F/52 | 0.99 | 0.98 | 35 | 38 | c.1078G>A | Het. | 46C/C | / |  |  |
| 14-Ⅲ6 | F/48 | 0.91 | 0.99 | 37 | 39 | c.1078G>A | Het. | 46C/C | / |  |  |
| 14-Ⅳ1 | M/28 | 1.00 | 1.91 | 11 | 10 | c.1078G>A | Homo. | 46C/C | / |  |  |
| 15-Ⅱ3 | F/26 | 0.95 | 1.95 | 12 | 13 | c.346G>A  c.1583C>A | Comp.Het | 46C/C | Antenatal care | 5/5 | NO |
| 15-Ⅰ1 | M/67 | 0.93 | 1.26 | 53 | 55 | c.346G>A | Het. | 46C/C | / |  |  |
| 15-Ⅰ2 | F/65 | 0.97 | 1.57 | 36 | 35 | c.1583C>A | Het. | 46C/C | / |  |  |
| 15-Ⅱ1 | F/33 | 0.99 | 1.23 | 50 | 51 | c.346G>A | Het. | 46C/C | / |  |  |
| 15-Ⅱ2 | M/30 | 0.94 | 1.48 | 35 | 34 | c.1583C>A | Het. | 46C/C | / |  |  |
| 16 | F/31 | 0.98 | 1.40 | 7 | 3 | c.1583C>A  c.1027G>C | Comp.Het | 46C/C | Neck mass | / | NO |
| 17 | M/24 | 0.96 | 3.40 | 4 | 2 | c.1681G>A | Homo. | 46T/T | Preoperative hemorrhoids | / | NO |
| 18 | F/18 | 1.00 | 4.44 | <1 | 1 | / | / | 46/CC | Preoperative appendicitis | / | / |
| 19 | M/60 | 0.97 | 3.27 | 4.8 | 3 | c.811-813delAAC▲ | Homo. | 46T/T | Gastritis | / | NO |
| 20-II1 | M/50 | 0.93 | 1.64 | 4 | 5 | c.1561G>A  c.1637T>C ▲ | Comp.Het | 46C/T | Preoperative fracture | 4/5 | NO |
| 20-I1 | M/70 | 0.90 | 1.31 | 30 | 32 | c.1637T>C | Het. | 46C/T | / |  |  |
| 20-I2 | F/69 | 0.98 | 1.02 | 34 | 33 | c.1561G>A | Het. | 46C/T | / |  |  |
| 20-II2 | F/50 | 1.05 | 1.06 | 98 | 101 | Wild | / | 46/CC | / |  |  |
| 20-III1 | F/25 | 1.08 | 1.27 | 32 | 31 | c.1561G>A | Het. | 46C/T | / |  |  |
| 21-Ⅱ1 | F/7 | 1.02 | 3.05 | 3 | 10 | c.1561G>A  c.1681G>A | Comp.Het | 46T/T | Preoperative shoulder lump | 4/4 | NO |
| 21-Ⅰ1 | M/35 | 1.09 | 1.05 | 54 | 49 | c.1681G>A | Het. | 46T/T | / |  |  |
| 21-Ⅰ2 | F/34 | 0.91 | 1.20 | 30 | 33 | c.1561G>A | Het. | 46C/T | / |  |  |
| 21-Ⅱ2 | M/4 | 0.92 | 1.23 | 35 | 37 | c.1681G>A | Het. | 46C/T | / |  |  |
| 22 | M/47 | 0.98 | 4.40 | 3 | 2 | c.1556T>C | Homo. | 46T/T | Chest distress | / | NO |
| 23 | M/58 | 0.85 | 3.20 | 5 | 1 | c.1681G>A | Homo. | 46C/C | Gastric ulcer | / | NO |
| 24 | M/54 | 0.99 | 1.78 | 7 | 3 | c.1078G>A | Homo. | 46C/C | Precordial pain | / | NO |
| 25 | M/60 | 1.03 | 2.02 | 3 | 1 | c.1078G>A | Homo. | 46C/C | Fatigue | / | NO |
| 26 | F/48 | 0.90 | 3.65 | 3 | 2 | c.1556T>C | Homo. | 46T/T | Preoperative adenomyosis | / | NO |
| 27-Ⅲ1 | F/24 | 0.90 | 3.94 | 5 | 2 | c.797G>A▲  c.810_812delCAA | Comp.Het | 46T/T | Drug overdose | 6/8 | NO |
| 27-Ⅰ1 | F/72 | 0.94 | 1.15 | 89 | 90 | wild | / |  | / |  |  |
| 27-Ⅰ2 | M/71 | 0.98 | 1.08 | 32 | 33 | c.797G>A | Het. |  | / |  |  |
| 27-Ⅱ1 | M/52 | 0.95 | 0.98 | 98 | 101 | wild | / |  | / |  |  |
| 27-Ⅱ2 | M/50 | 0.97 | 1.19 | 34 | 36 | c.797G>A | Het. |  | / |  |  |
| 27-Ⅱ3 | F/48 | 0.91 | 1.18 | 31 | 30 | c.797G>A | Het. |  | / |  |  |
| 27-Ⅱ4 | M/50 | 1.00 | 1.02 | 33 | 33 | c.810_812delCAA | Het. |  | / |  |  |
| 27-Ⅲ2 | F/21 | 0.94 | 1.15 | 32 | 31 | c.797G>A | Het. |  | / |  |  |
| 28 | F/46 | 0.90 | 4.98 | 3 | 2 | c.78_78delC▲  c.1027G>C  c.1681G>A | Comp.Het | 46T/T | Fatigue | / | NO |
| 29-Ⅱ1 | M/54 | 0.92 | >5 | 1 | 1 | c.797G>A  c.1681G>A | Comp.Het | 46T/T | Cough | 5/6 | NO |
| 29-Ⅰ1 | M/77 | 0.98 | 1.26 | 43 | 48 | c.797G>A | Het. | 46C/T | / |  |  |
| 29-Ⅱ2 | F/52 | 0.97 | 0.91 | 102 | 98 | wild | / | 46C/C | / |  |  |
| 29-Ⅲ1 | M/30 | 1.05 | 1.31 | 45 | 43 | c.797G>A | Het. | 46C/T | / |  |  |
| 29-Ⅲ2 | F/27 | 0.97 | 1.23 | 53 | 54 | c.1681G>A | Het. | 46C/T | / |  |  |
| 29-Ⅲ3 | M/25 | 0.96 | 1.04 | 51 | 57 | c.1681G>A | Het. | 46C/T | / |  |  |
| 30-Ⅳ1 | M/58 | 0.93 | 2.81 | <1 | 1 | c.1638G>A▲ | Homo. | 46C/C | Gastritis | 4/5 | YES |
| 30-III2 | F/80 | 0.97 | 1.27 | 45 | 50.1 | c.1638G>A | Het. | 46C/C | / |  |  |
| 30-Ⅳ2 | F/57 | 0.98 | 1.01 | 98 | 103.2 | wild | / | 46C/C | / |  |  |
| 30-V1 | M/30 | 0.93 | 1.28 | 41 | 45.1 | c.1638G>A | Het. | 46C/C | / |  |  |
| 30-V2 | F/26 | 0.99 | 1.37 | 38 | 43.1 | c.1638G>A | Het. | 46C/C | / |  |  |
| 31-III2 | F/25 | 1.01 | 4.79 | 3 | 2 | c.1092_1093insC | Het. | 46T/T | Trauma | 3/8 | NO |
| 31-I2 | F/74 | 0.97 | 1.59 | 21 | 23.5 | c.1092_1093insC | Het. | 46C/T | / |  |  |
| 31-I3 | M/73 | 1.00 | 1.09 | 72 | 77.6 | wild | / | 46C/T | / |  |  |
| 31-I4 | F/75 | 0.94 | 1.17 | 54 | 53.5 | wild | / | 46T/T | / |  |  |
| 31-II1 | M/53 | 0.99 | 1.64 | 18 | 20.2 | c.1092_1093insC | Het. | 46C/T | / |  |  |
| 31-II2 | F/50 | 1.01 | 1.15 | 61 | 66.1 | wild | / | 46T/T | / |  |  |
| 31-III1 | F/28 | 0.97 | 1.06 | 79 | 84.5 | wild | / | 46C/T | / |  |  |
| 31-III3 | M/23 | 1.03 | 1.16 | 65 | 69.6 | wild | / | 46T/T | / |  |  |
| 32-II1 | M/42 | 0.90 | 3.61 | 5 | 1 | c.1561G>A  c.1092_1093insC | Comp.Het | 46C/T | Preoperative bile duct stone | 5/6 | NO |
| 32-I1 | M/67 | 0.98 | 1.14 | 44 | 40 | c.1561G>A | Het. | 46C/T | / |  |  |
| 32-I2 | F/65 | 0.94 | 1.31 | 38 | 36 | c.1092_1093insC | Het. | 46C/T | / |  |  |
| 32-II2 | F/42 | 0.97 | 0.90 | 92 | 100 | wild | / | 46C/C | / |  |  |
| 32-III1 | F/19 | 1.05 | 1.31 | 36 | 39 | c.1092_1093insC | Het. | 46C/T | / |  |  |
| 32-III2 | M/15 | 1.00 | 1.14 | 37 | 35 | c.1561G>A | Het. | 46C/T | / |  |  |
| 33-Ⅳ3 | F/27 | 1.04 | >5 | 3 | 5 | c.1748T>A▲ | Homo. | 46C/C | Preoperative shoulder injury | 5/7 | YES |
| 33-Ⅲ1 | F/60 | 0.97 | 2.42 | 52 | 58 | c.1748T>A | Het. | 46C/C | / |  |  |
| 33-Ⅲ2 | M/59 | 1.00 | 2.36 | 56 | 51 | c.1748T>A | Het. | 46C/C | / |  |  |
| 33-Ⅳ1 | M/32 | 0.95 | 2.11 | 48 | 50 | c.1748T>A | Het. | 46C/C | / |  |  |
| 33-Ⅳ2 | M/30 | 0.96 | 0.97 | 92 | 101 | wild | / | 46C/C | / |  |  |
| 33-Ⅳ4 | M/29 | 1.04 | 1.08 | 80 | 97 | wild | / | 46C/C | / |  |  |
| 33-V1 | M/3 | 1.01 | 2.08 | 45 | 44 | c.1748T>A | Het. | 46C/C | / |  |  |
| 34-II1 | M/47 | 1.02 | >5 | <1 | <1 | c.1792_1796del5bp▲  c.1092-1093insC | Comp.Het | 46T/T | Gastritis | 6/7 | NO |
| 34-I1 | M//70 | 0.92 | 1.18 | 46 | 50.3 | c.1792_1796del5bp | Het. | 46C/T | / |  |  |
| 34-I2 | F/69 | 0.95 | 1.2 | 39 | 43.1 | c.1092-1093insC | Het. | 46C/T | / |  |  |
| 34-II2 | F/45 | 0.98 | 1.04 | 78 | 82.1 | wild | / | 46C/T | / |  |  |
| 34-II3 | M/45 | 0.97 | 1.12 | 43 | 47.5 | c.1792_1796del5bp | Het. | 46C/T | / |  |  |
| 34-III1 | M/22 | 1.05 | 1.71 | 16 | 18.8 | c.1092-1093insC | Het. | 46T/T | / |  |  |
| 34-III2 | M/19 | 0.95 | 1.6 | 24 | 20.1 | c.1092-1093insC | Het. | 46T/T | / |  |  |
| 35-Ⅳ2 | M/51 | 0.91 | >5 | <1 | <1 | c.1A>G | Homo. | 46C/C | Preoperative kidney stones | 5/6 | YES |
| 35-Ⅲ2 | M/72 | 0.88 | 1.33 | 28 | 30.2 | c.1A>G | Het. | C/T | / |  |  |
| 35-Ⅲ1 | F/75 | 0.88 | 1.36 | 25 | 33.6 | c.1A>G | Het. | C/T | / |  |  |
| 35-Ⅳ1 | F/52 | 0.98 | 1.09 | 68 | 63.4 | wild | / | T/T | / |  |  |
| 35-Ⅳ3 | F/49 | 0.94 | 1.22 | 31 | 29.8 | c.1A>G | Het. | C/T | / |  |  |
| 35-Ⅴ1 | M/28 | 0.88 | 1.21 | 36 | 40.1 | c.1A>G | Het. | C/T | / |  |  |
| 36 | M/54 | 1.06 | >5 | <1 | <1 | / | / | 46T/T | Preoperative hemorrhoids | / | NO |
| 37-Ⅱ1 | F/54 | 1.13 | >5 | 3 | 2.1 | c.712_713insT▲  c.1561G>A | Comp.Het | 46C/T | Preoperative ovarian cyst | 4/5 | NO |
| 37-I1 | F/74 | 0.92 | 1.13 | 42 | 43.6 | c.712_713insT | Het. | 46C/T | / |  |  |
| 37-Ⅱ2 | M/56 | 0.97 | 0.98 | 88 | 91.4 | wild | / | 46C/T | / |  |  |
| 37-Ⅲ1 | M/27 | 1.03 | 1.11 | 45 | 49.7 | c.1561G>A | Het. | 46C/C | / |  |  |
| 37-Ⅲ2 | M/25 | 0.88 | 1.03 | 39 | 40.6 | c.712_713insT | Het. | 46C/T | / |  |  |
| 38 | F/33 | 1.01 | 4.06 | 3 | 2 | c.1092_1093insC | Homo. | 46T/T | Infertility | / | NO |
| 39-Ⅱ2 | F/51 | 0.97 | >5 | 1 | 1 | c.218G>C  c.303_304delCA | Comp.Het | 46T/T | Preoperative kidney stones | 2/3 | NO |
| 39-Ⅱ1 | M/53 | 1.01 | 1.08 | 86 | 90 | wild | / | 46C/T | / |  |  |
| 39-Ⅲ1 | M/26 | 0.99 | 1.66 | 19 | 24 | c.303_304delCA | Het. | 46T/T | / |  |  |
| 40 | F/44 | 0.95 | 1.88 | 8 | 3 | c.1078G>A | Het. | 46C/T | Check up | / | NO |
| 41 | F/47 | 0.95 | >5 | 3 | 2 | c.301-302delCA | Homo. | 46C/C | Preoperative kidney stones | / | NO |
| 42 | M/46 | 0.93 | >5 | <1 | 1 | c.799C>G | Homo. | 46C/C | Check up | / | NO |
| 43-Ⅱ7 | F/37 | 1.13 | 5.00 | 3 | 1 | c.1561G>A | Het. | 46T/T | Trauma | 3/8 | NO |
| 43-I1 | M/72 | 0.89 | 1.08 | 92 | 99.5 | wild | / | 46C/T | / |  |  |
| 43-I2 | F/69 | 0.93 | 1.38 | 56 | 60.2 | wild | / | 46T/T | / |  |  |
| 43-Ⅱ5 | F/43 | 0.81 | 1.40 | 47 | 50.5 | wild | / | 46T/T | / |  |  |
| 43-Ⅲ1 | F/27 | 1.28 | 5 | 3 | 2 | c.1561G>A | Het. | 46T/T | / |  |  |
| 43-Ⅲ2 | M/23 | 1.31 | 4.45 | 3 | 1 | c.1561G>A | Het. | 46T/T | / |  |  |
| 43-Ⅲ3 | M/23 | 1.29 | 1.56 | 47 | 50.5 | wild | / | 46T/T | / |  |  |
| 43-Ⅲ5 | M/16 | 1.43 | 1.54 | 40 | 46.3 | wild | / | 46T/T | / |  |  |
| 44-Ⅳ1 | F/35 | 1.01 | 2.13 | 3 | 4 | c.809_811delACA | Homo. | 46C/C | Post-natal check | 4/5 | YES |
| 44-III1 | M/61 | 1.01 | 1.58 | 34 | 33 | c.809_811delACA | Het. | 46C/C | / |  |  |
| 44-III2 | F/59 | 0.94 | 1.53 | 35 | 36 | c.809_811delACA | Het. | 46C/C | / |  |  |
| 44-Ⅳ2 | F/33 | 0.97 | 0.79 | 103 | 102 | wild | / | 46C/C | / |  |  |
| 44-Ⅳ3 | M/30 | 0.92 | 1.44 | 31 | 29 | c.809_811delACA | Het. | 46C/C | / |  |  |
| 45-II2 | M/68 | 0.90 | 2.86 | 2 | 2 | c.1556T>G | Homo. | 46C/C | Diabetes | 5/6 | YES |
| 45-II1 | F/70 | 0.89 | 1.16 | 56 | 49 | c.1556T>G | Het. | 46C/C | / |  |  |
| 45-II3 | F/66 | 0.90 | 0.96 | 124 | 110 | wild | / | 46C/C | / |  |  |
| 45-III1 | F/40 | 0.93 | 1.09 | 42 | 48 | c.1556T>G | Het. | 46C/C | / |  |  |
| 45-III2 | F/37 | 0.98 | 1.13 | 48 | 46 | c.1556T>G | Het. | 46C/C | / |  |  |
| 45-Ⅳ1 | M/10 | 0.97 | 1.16 | 38 | 36 | c.1556T>G | Het. | 46C/T | / |  |  |
| 46-Ⅳ1 | F/74 | 1.00 | 3.04 | 3 | 2 | c.566G>C | Homo. | 46C/C | Preoperative thyroid nodule | 4/5 | YES |
| 46-Ⅲ2 | F/92 | 1.01 | 1.16 | 34.0 | 33.0 | c.566G>C | Het. | 46C/C | / |  |  |
| 46-Ⅳ2 | M/76 | 0.95 | 1.11 | 104.0 | 102.0 | wild | / | 46C/C | / |  |  |
| 46-Ⅴ1 | M/50 | 0.97 | 1.2 | 32.0 | 30.0 | c.566G>C | Het. | 46C/C | / |  |  |
| 46-Ⅴ2 | F/48 | 0.94 | 1.14 | 39.0 | 37.0 | c.566G>C | Het. | 46C/C | / |  |  |
| 47-Ⅲ1 | M/24 | 0.95 | 4.94 | 3 | 4 | c.130delG▲  c.1561G>A | Comp.Het | 46C/C | Facial neuritis | 4/4 | NO |
| 47-I2 | F/70 | 1.04 | 1.22 | 40 | 38 | c.130delG | Het. | 46C/C | / |  |  |
| 47-II2 | M/48 | 0.97 | 1.26 | 38 | 37 | c.130delG | Het. | 46C/C | / |  |  |
| 47-Ⅲ2 | M/22 | 0.96 | 1.23 | 39 | 40 | c.130delG | Het. | 46C/C | / |  |  |
| 48 | F/25 | 1.01 | 4.22 | 1 | 1 | c.809-811delACA | Het. | 46T/T | Infertility | / | NO |
| 49 | F/34 | 0.92 | 2.20 | 5 | 2 | c.1092_1093insC | Het. | 46C/T | Infertility | / | NO |
| 50-II3 | M/47 | 1.25 | 4.87 | 3 | 1 | c.1561G>A | Het. | 46C/T | Preoperative kidney stones | 4/6 | NO |
| 50-II1 | F/80 | 1.14 | 1.81 | 19 | 15.5 | c.1561G>A | Het. | 46C/T | / |  |  |
| 50-II2 | F/57 | 1.21 | 1.82 | 19 | 20.2 | c.1561G>A | Het. | 46C/T | / |  |  |
| 50-Ⅲ2 | F/18 | 1.13 | 1.56 | 45 | 48.8 | wild | / | 46T/T | / |  |  |
| 50-Ⅲ3 | M/15 | 1.34 | 2.01 | 15 | 13 | c.1561G>A | Het. | 46C/T | / |  |  |
| 50-Ⅲ4 | F/12 | 1.40 | 1.43 | 39 | 42.3 | wild | / | 46T/T | / |  |  |
| 51 | M/26 | 1.14 | >5 | <1 | 1 | / | / | 46T/T | Preoperative tooth extraction | / | NO |
| Range | / | 0.85-1.15 | 0.8-1.20 | 72-113 | 72-113 | / | / | / | / | / | / |

▲: The mutation sites that our team initially discovered; P:proband; G/A: Gender/Age; M:male; F:female;INR:international normalized ratio; APTTR:activated partial thromboplastin time ratio

FXII:C:factor FXII activity; Het:heterozygote; Homo:homozygote; Comp.Het:compound heterozygote
